# Application of PLASMIC Score in Risk Prediction of Thrombotic Thrombocytopenic Purpura: Real-World Experience From a Tertiary Medical Center in Taiwan

**DOI:** 10.3389/fmed.2022.893273

**Published:** 2022-05-09

**Authors:** Chun-Hui Lee, Yi-Ching Huang, Sin-Syue Li, Ya-Ting Hsu, Ya-Ping Chen, Tsai-Yun Chen

**Affiliations:** ^1^Institute of Clinical Medicine, College of Medicine, National Cheng Kung University, Tainan, Taiwan; ^2^Department of Oncology, National Cheng Kung University Hospital, College of Medicine, National Cheng Kung University, Tainan, Taiwan; ^3^Division of Hematology, Department of Internal Medicine, National Cheng Kung University Hospital, College of Medicine, National Cheng Kung University, Tainan, Taiwan; ^4^Center for Cell Therapy, National Cheng Kung University Hospital, College of Medicine, National Cheng Kung University, Tainan, Taiwan

**Keywords:** thrombotic thrombocytopenic purpura, thrombotic microangiopathy, ADAMTS13 (a disintegrin-like and metalloprotease with thrombospondin type 1 repeats), PLASMIC score, relapsed, refractory, systemic lupus erythematosus

## Abstract

Thrombotic thrombocytopenic purpura (TTP) is a life-threatening disorder caused by severe ADAMTS13 (a disintegrin and metalloprotease with thrombospondin type 1 repeats, member 13) deficiency (activity <10%). Urgent intervention based on the timely evaluation of ADAMTS13 level is crucial to guide optimal therapy. The recently developed PLASMIC score based on seven items allows the rapid identification of patients at high risk for TTP due to severe ADAMTS13 deficiency. This retrospective study included 31 hospitalized patients with suspicious thrombotic microangiopathy in National Cheng Kung University Hospital from December 2016 to July 2021. Data on ADAMTS13 activity and medical and laboratory information were retrieved from medical records. The PLASMIC score could be calculated in 24 of the 31 patients with available data, and the final cohort was stratified according to the 7-point PLASMIC score. All patients with high PLASMIC score (6–7) exhibited severe ADAMTS13 deficiency (activity ≤10%). One patient with a brain tumor and a PLASMIC score of 6 did not have severe ADAMTS13 activity of ≤10%. The patients in the intermediate- and low risk groups (PLASMIC scores of 5 and 0–4, respectively) exhibited ADAMTS13 activities of above 10%. Given the role of prompt diagnosis in the timely delivery of appropriate therapy, these findings confirm and strengthen the predictive value of the PLASMIC score in patients at high risk for TTP due to severe ADAMTS13 deficiency.

## Introduction

Thrombotic thrombocytopenic purpura (TTP), first described by Moschcowitz in 1924 (1), is a life-threatening disease with a mortality rate of 10–20% despite appropriate therapeutic management (2). TTP is a rare form of thrombotic microangiopathy (TMA) characterized by microangiopathic hemolytic anemia (MAHA) accompanied with severe thrombocytopenia and organ ischemia secondary to disseminated microvascular platelet-rich thrombi. For a particular underlying cause, the clinical features observed in patients with TMA are neither sensitive nor specific. Therefore, rigorously derived and easily deployable clinical diagnostic tools that can identify individuals with TTP are critical.

In 1998, deficiency of ADAMTS13 (a disintegrin and metalloprotease with thrombospondin type 1 repeats, member 13), a von Willebrand factor-cleaving protease, has been recognized as the cause of TTP (3–5). Since then, several studies in patients with TMA have demonstrated that an ADAMTS13 activity below 10% is a specific feature of TTP. The diagnosis of TTP requires prompt attention given that it is a fatal condition that requires urgent plasmapheresis. However, testing for ADAMTS13 activity is not widely available and has long turnaround times.

In the last two decades, major advances have facilitated our understanding of TTP and ADAMTS13. Several clinical scoring systems have been developed for the rapid identification of patients who are most likely to have severe ADAMTS13 deficiency. These diagnostic scores have been evaluated for their ability to improve diagnostic accuracy and to guide early treatment in patients with TTP (6–9). In 2010, Bentley et al. published the first clinical diagnostic tool for TTP. Total of five parameters (platelet count, D-dimer, reticulocytes, creatinine, and indirect bilirubin) were found to be predictive of severe ADAMTS13 deficiency (10). The French score with three-components, platelet count, creatinine and antinuclear antibody, described by Coppo et al. in 2010, is an alternative scoring system used to identify TTP (11). Encouragingly, the PLASMIC score, which was derived based on the data of 214 patients in the multi-institutional Harvard TMA Research Collaborative registry, was able to predict severe ADAMTS13 deficiency. The PLASMIC score is used to stratify patients with TMA according to their risk of severe ADAMTS13 deficiency based on the following seven items: platelet count <30 × 10^9^/L, hemolysis variable (elevated reticulocyte count, undetectable haptoglobin, or indirect bilirubin >2.0 mg/dL), no active cancer, no history of solid organ or stem cell transplant, mean corpuscular volume (MCV) <90 fL, international normalized ratio (INR) <1.5, and creatinine <2.0 mg/dL (7, 12, 13).

Therapeutic plasmapheresis (TPE) remains the cornerstone of TTP management (14, 15). Plasmapheresis, which usually starts as a 1.5-fold plasma volume exchange followed by a 1.0-fold plasma volume exchange thereafter, should be commenced immediately in patients undergoing diagnostic workup for suspicious TTP. Plasmapheresis is performed daily until the resolution of clinical manifestations related to organ involvement, stable recovery of platelet counts, and cessation of hemolysis (16).

Thrombotic thrombocytopenic purpura is a medical emergency, and its prompt recognition is imperative because of the high mortality rates in untreated or mismanaged patients. Disparities in early diagnosis and timely treatment remain a clinical unmet need.

## Materials and Methods

### Study Cohort

**FIGURE 1:**
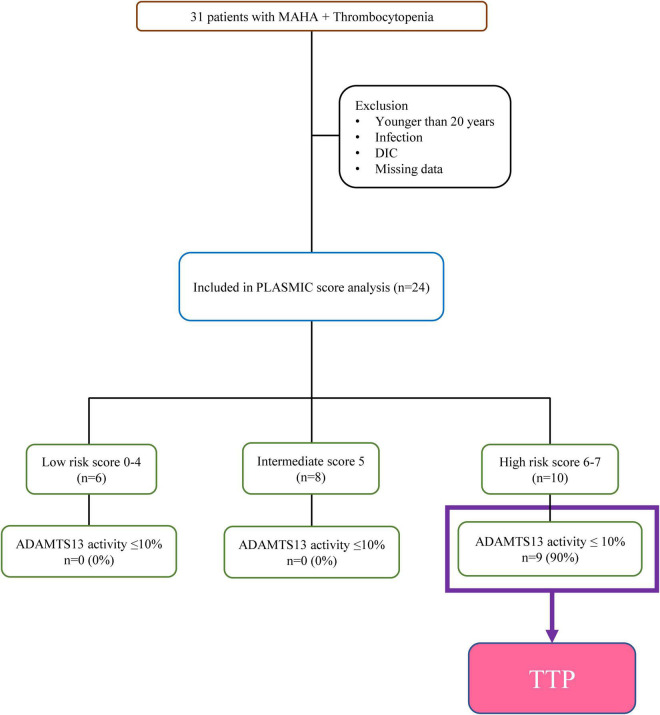
Flow chart describing patients from December 2016 to July 2021.

This retrospective cohort included adult patients aged ≥20 years who were evaluated for TMA at National Cheng Kung University Hospital in Taiwan between December 2016 and July 2021. The diagnosis of TMA was based on the presence of thrombocytopenia (platelet count <150 × 10^9^/L) and MAHA (hemoglobin <10 g/dL in the presence of schistocytes). Patients with documented infection or disseminated intravascular coagulation were excluded. Patients with no laboratory data required before plasmapheresis and those with missing data were also excluded (Figure 1).

Patients were categorized as those with TTP and those with other forms of TMA. The diagnosis of TTP was based on a documented ADAMTS13 activity of ≤10%. All patients who did not meet the diagnostic criteria for TTP were classified as those with other forms of TMA, including atypical hemolytic uremic syndrome, systemic lupus erythematosus (SLE) associated TMA, drug-induced TMA, transplant-associated TMA, malignant hypertension-associated TMA, malignancy-associated TMA, and unexplained TMA. Relapsed TTP that was defined as disease recurrence after ≥30 days since TPE discontinuation (17). In patients who were refractory, it was defined as a failure of platelet response after 4–7 days of TPE or a clinical deterioration in a patient receiving standard therapy (18).

The ADAMTS13 activity was performed using a chromogenic enzyme-linked immunosorbent assay (TECHNOZYM^®^ ADAMTS-13 activity ELISA; Technoclone). In the study institution, a hematology consult was required in cases where TMA was considered and ADAMTS13 activity could be determined only in patients with suspicious TMA.

### Data Collection and Risk Stratification

The clinical and laboratory information were retrieved from the medical records. Data included age, sex, initial presentation, comorbidities, and treatment course. The study patients were stratified according to the 7-point PLASMIC score, which included platelet count <30 × 10^9^/L, hemolysis variable (elevated reticulocyte count, undetectable haptoglobin, or indirect bilirubin >2.0 mg/dL), no active cancer, no history of solid organ or stem cell transplant, MCV <90 fL, INR <1.5, and creatinine <2.0 mg/dL. The presence of each item is worth 1 point, with a maximum PLASMIC score of 7. The PLASMIC scores of 0–4, 5, and 6–7 indicate low, intermediate, and high risk for severe ADAMTS13 deficiency, respectively (7). The level of agreement between the PLASMIC score and ADAMTS13 activity was evaluated. To verify the predictive value of severe acquired ADAMTS13 deficiency at the time of diagnosis, the French score, which involves three items (platelet count ≤30 × 10^9^/L, serum creatinine ≤2.26 mg/dL, and antinuclear antibody positivity), was also calculated. The presence of each item in the French score is worth 1 point, and French scores of 0, 1, and 2–3 indicate low, intermediate, and high risk of severe ADAMTS13 deficiency, respectively (11). The study was approved by the Institutional Review Board at the National Cheng Kung University Hospital (IRB no., B-ER-110-256).

### Statistical Analysis

GraphPad Prism statistical software (version 9; GraphPad, San Diego, CA, United States) was used for all statistical analyses. Continuous data were presented as means ± SD or medians (interquartile range) depending on the distribution. Categorical data were presented as numbers and percentages. Comparisons of normally and abnormally distributed continuous variables were performed using Student’s *t* and the Mann–Whitney *U* tests, respectively.

## Results

During the study period, 24 of the 31 consecutive patients who were evaluated fulfilled the criteria for TMA (Figure 1). The ADAMTS13 activity levels were ≤10 and >10% in 9 and 15 patients with TMA, respectively. The PLASMIC score could be determined in all 24 patients (100%) who had data available for all seven items, and nine of the 24 patients with TMA were diagnosed with TTP according to the ADAMTS13 activity results.

Table 1 summarizes the clinical and laboratory characteristics and the treatment course of the patients with TTP and those with other forms of TMA (non-TTP TMA). Briefly, sex (*P* = 0.9325), age (*P* = 0.4369), hemoglobin (*P* = 0.7585), INR (*P* = 0.4102), and lactate dehydrogenase (*P* = 0.1737) were not significantly different between the patients with TTP and those with other TMA. Conversely, the patients with TTP had significantly higher MCV (*P* = 0.0239), lower platelet count (*P* = 0.0007), and lower creatinine (*P* = 0.0017) compared with other TMA. Because of the severity of symptoms and the need for plasmapheresis, all patients with TTP underwent more cycles of plasmapheresis than those with other TMA (median, 14 versus 3 days; *P* < 0.0001) (Table 1).

**TABLE 1:** Characteristics of patients with TTP and those with other forms of TMA.

	TTP (*n* = 9)	Other TMA (*n* = 15)	*P*-value
Female sex	7 (77.8%)	12 (80%)	0.9325
Age, median (range)	46 (22–70)	42(19–87)	0.4369
ADAMTS13 activity, median, % (range)	0	49 (20–86)	<0.0001
Laboratory, median (range)			
Hemoglobin count (g/dL)	7.2 (5.4–9.9)	7.1 (5.3–10.3)	0.7585
MCV (fL)	90.9 (82.4–97.9)	87.2 (71.8–92.8)	0.0239
INR	1.08 (1.02–1.12)	1.19 (0.82–2.26)	0.4102
Platelet (10^3^/μL)	1.1 (4–21)	5.7 (13–105)	0.0007
Creatinine (mg/dL)	0.69 (0.44–1.25)	4.27 (0.32–16.49)	0.0017
Lactate dehydrogenase (U/L)	930 (352–5065)	608 (373–2853)	0.1737
Plasmapheresis, *n* (%)	9 (100)	9 (60)	
Days of plasmapheresis			
Median days (range)	14 (8–25)	3 (0–19)	<0.0001
Immunosuppressant, *n* (%)	9 (100)	11 (73)	
Glucocorticoids	9 (100)	9 (60)	
Rituximab	4 (45)	3 (20)	
Cyclophosphamide	2 (22)	0	
Other treatments, *n* (%)			
Eculizumab	0	1 (7)	
Eltrombopag	0	1 (7)	

*TTP, thrombotic thrombocytopenic purpura; TMA, thrombotic microangiopathy; ADAMTS13, a disintegrin and metalloprotease with thrombospondin type 1 repeats, member 13; MCV, mean corpuscular volume; INR, international normalized ratio.*

The PLASMIC score was used to stratify the entire study cohort (*n* = 24) into three risk categories (Table 2). The diagnosis of TTP based on an ADAMTS13 activity of ≤10% was confirmed in nine of the ten patients stratified into the high risk group (PLASMIC score, 6 or 7). The positive predictive value of the PLASMIC score was 90% (95% confidence interval, 57.53–98.35%). The intermediate risk group (PLASMIC score, 5) included one patient with atypical hemolytic uremic syndrome, four patients with SLE-associated TMA, one patient with malignant hypertension, and two patients with unexplained TMA. The low risk group (PLASMIC score, 0–4) included one patient with SLE-associated TMA, two patients with drug-induced TMA, and two patients with transplant-associated TMA. The negative predictive value of the PLASMIC score was 100% (Supplementary Table 1).

**TABLE 2:** PLASMIC score, ADAMTS13 activity, and distribution of clinical diagnoses.

	PLASMIC score risk prediction (*n* = 24)		
	Low risk*^a^* (*n* = 6)	Intermediate risk*^b^* (*n* = 8)	High risk*^c^* (*n* = 10)
ADAMTS13 activity ≤10%, *n* (%)	0	0	9 (90)
ADAMTS13 activity >10%, *n* (%)	6 (100)	8 (100)	1 (10)
Clinical diagnosis, *n* (%)			
TTP	0	0	9 (90)
Other TMA			
aHUS	0	1 (12.5)	0
SLE-associated TMA	1 (16.7)	4 (50)	0
DI-TMA	2 (33.3)	0	0
TA-TMA	2 (33.3)	0	0
Malignant hypertension	0	1 (12.5)	0
Unexplained TMA	1(16.7)	2 (25)	0
Malignancy	0	0	1(10)

*^a^Low risk: PLASMIC score of 0–4.*

*^b^Intermediate risk: PLASMIC score of 5.*

*^c^High risk: PLASMIC score of 6–7.*

*ADAMTS13, a disintegrin and metalloprotease with thrombospondin type 1 repeats, member 13; TTP, thrombotic thrombocytopenic purpura; TMA, thrombotic microangiopathy; aHUS, atypical hemolytic uremic syndrome; SLE, systemic lupus erythematosus; DI-TMA, drug-induced thrombotic microangiopathy; TA-TMA, transplant-associated thrombotic microangiopathy.*

In the present study cohort, the positive predictive value of the French score, a simpler ADAMTS13 deficiency risk prediction tool, was 64.3%, which was lower than that of the PLASMIC score (Supplementary Table 2).

The median follow-up period was 18.5 months (range, 1–44 months). There was no mortality during the acute phase after TMA presentation in the TTP group. Nevertheless, three patients (patient nos. 1, 4, and 5) developed relapsed or refractory TTP (Table 3). These patients initially presented with neurological deficits, and patient no. 4 recovered after another cycle of consecutive plasmapheresis. Patient nos. 1 and 5 had a refractory disease and received anti-CD20 monoclonal antibody (rituximab) and other immunosuppressants in addition to the therapeutic plasma exchange.

**TABLE 3:** Baseline characteristics and serial ADAMTS13 activity levels of patients with TTP.

No.	Sex	Age	Comorbidity	Initial presentation	PLASMIC score	ADAMTS13 (%)*	Relapsed/ refractory	ADAMTS13 (%)_DOF	ADAMTS13 (%)_DOF
1	F	51	Nil	Altered consciousness	6	0	Yes	0 4 days	0 15 days
2	F	40	Nil	Petechia	7	0	No		
3	F	22	Nil	Syncope	6	0	No		
4	M	70	T2DM HTN	Drowsiness	6	0	Yes	0 106 days	
5	F	33	SLE	Dizziness	7	0	Yes	0 57 days	24 384 days
6	M	61	HBV	Dizziness	6	0	No		
7	F	34	SLE	Headache	7	0	No		
8	F	62	HTN	Headache	6	0	No		
9	F	37	SLE	Petechia	6	0	No		

ADAMTS13, a disintegrin and metalloprotease with thrombospondin type 1 repeats, member 13; TTP, thrombotic thrombocytopenic purpura; T2DM, type 2 diabetes mellitus; HTN, hypertension; SLE, systemic lupus erythematosus; HBV, chronic hepatitis B infection; ADAMTS13(%)*: ADAMTS13 activity level at presentation; DOF: days of follow-up after first ADAMTS13 activity result.

In the high risk group, one of the 10 patients, a 38-year-old woman with ADAMTS13 activity level of >10%, initially presented with intermittent headache; the image showed brain tumor with hemorrhage. She exhibited an immediate drop in platelet count with MAHA, during the hospitalization. The clinical course was fulminant, and she eventually succumbed to septic shock.

## Discussion

Timely identification of patients with TMA who require urgent treatment is a key unmet clinical need. The retrospective analysis of the current cohort of 24 patients based on risk stratification using the 7-point PLASMIC score confirmed the role of clinical assessment in the timely detection of severe ADAMTS13 deficiency in adult patients with TMA. In agreement with previous studies (8, 19, 20), none of the patients with low risk had severe ADAMTS13 deficiency. Additionally, 90% of the high risk patients with severe ADAMTS13 deficiency benefited from prompt therapeutic plasmapheresis. These patients received plasmapheresis and glucocorticoids treatment immediately once clinical judgment supports the diagnosis of TTP.

The French score, described by Coppo et al., is an alternative scoring system used to identify TTP (11). In the present study, the PLASMIC score with internal and external validation by the Harvard TMA registry, exhibiting superior performance in the prediction of ADAMTS13-deficient TTP compared with the French score (Supplementary Tables 1, 2). Data on antinuclear antibody levels were not collected at the initial presentation in most cases. Moreover, differences were observed in the TMA patient selection between the French and PLASMIC scores. The French Score relies on an increased level of clinical judgment from the provider to employ, whereas the PLASMIC score is intended for all-comers even when TTP does not have as high a degree of suspicion that the French score assumes at baseline. A recent brief report demonstrated a moderate correlation among three clinical TMA diagnostic scoring systems (PLASMIC, French, Bentley) and ADAMTS13 levels (21). Considering these instruments, two important caveats are identified: the exact definition of severe ADAMTS13 deficiency remains unclear, which may be assay dependent, and the cutoffs applied to generate these score systems varied across studies; no prospective research has been conducted on these prediction tools (22).

Acquired TTP, which is immunologically mediated, is associated with several autoimmune disorders such as SLE and Hashimoto’s thyroiditis. The diagnosis of TTP in the setting of SLE may be difficult because of overlapping clinical symptoms. Likewise, the laboratory criteria of MAHA, which include the presence of schistocytes in the peripheral blood smear, are extremely subjective and rely on experienced laboratorians and hematologists. In the present study, 3 of the 10 patients (30%) in the high risk group were definitively diagnosed with ADAMTS13-deficient TTP associated with SLE. Patients with SLE are at a higher risk for SLE-associated TTP-like MAHA. The underlying pathophysiology is frequently heterogeneous and can be secondary to antiphospholipid syndrome or vasculitis (23). Five of the patients with TMA in the intermediate- and low risk groups had concurrent SLE. In a study of 1,203 SLE cases in Korea, Kwok et al. reported that 2.2% of the patients presented with the clinical features of TTP at the time of diagnosis (24). In two other studies including 40 and 105 patients, concurrent SLE was present in 12 and 45% of the patients with TTP, respectively (25, 26). Moreover, a review of the literature encompassing the period from 1968 to 2002 reported that the mortality was higher among 56 patients with concurrent SLE and TTP compared with those with idiopathic TTP despite optimal treatment (27).

Relapsed TTP is defined as disease recurrence after ≥30 days since TPE discontinuation (17). Refractory TTP is defined as a failure of platelet response after 4–7 days of plasmapheresis or a clinical deterioration despite standard therapy (18). In patients with relapsed or refractory TTP despite treatment with ADAMTS13 inhibitors, patient reevaluation is important to identify other potential etiologies of MAHA and thrombocytopenia. In the present cohort, three of the nine patients with TTP (34%) experienced relapsed or refractory TTP and one of these patients received 100 mg rituximab once and exhibited a good response. Emerging evidence suggests that weekly pulse rituximab can achieve a good response in patients with relapsed or refractory TTP (28–30).

The study cohort utilized for the derivation of the PLASMIC score was from the Harvard TMA registry, representing mostly Western ethnic groups (7). In their study, Tiscia et al. demonstrated the good diagnostic performance of the PLASMIC score in a cohort from Southern Italy (19). Another study of a small Chinese cohort reported that a modified PLASMIC score, which included lactate dehydrogenase in addition to the original seven items of the PLASMIC scoring system, might be more suitable for identifying patients with ADAMTS13 deficiency (31). To the best of our knowledge, few studies with larger cohorts have validated the PLASMIC score as a new prediction tool and this is the first study to verify the ability of the PLASMIC score to predict TTP risk in an East Asian cohort.

The present study has some limitations that should be acknowledged. First, this was a retrospective study; however, this approach was unavoidable because of the rarity of TMA with severe ADAMTS13 deficiency. Second, the cohort size was small because of the low rate of TTP.

Albeit rare, acquired TTP is associated with high mortality because of an aggressive clinical course in the setting of delayed diagnosis without optimal treatment. The present study results support the utility of the PLASMIC score as a rapid, feasible, and reliable clinical assessment tool to predict severe ADAMTS13 deficiency in adult patients with TMA.

## Data Availability Statement

The original contributions presented in the study are included in the article/Supplementary Material, further inquiries can be directed to the corresponding author/s.

## Author Contributions

C-HL designed and conducted the study, collected the data, performed the statistical analyses, and interpreted and wrote the manuscript. Y-CH conducted the study and collected the data. S-SL, Y-TH, and Y-PC conducted the study. T-YC designed the study, analyzed and interpreted the data, and wrote the manuscript. All authors contributed to the article and approved the submitted version.

## Conflict of Interest

The authors declare that the research was conducted in the absence of any commercial or financial relationships that could be construed as a potential conflict of interest.

## Publisher’s Note

All claims expressed in this article are solely those of the authors and do not necessarily represent those of their affiliated organizations, or those of the publisher, the editors and the reviewers. Any product that may be evaluated in this article, or claim that may be made by its manufacturer, is not guaranteed or endorsed by the publisher.
