# Supplementary material for: Application of PLASMIC Score in Risk Prediction of Thrombotic Thrombocytopenic Purpura: Real-World Experience From a Tertiary Medical Center in Taiwan
Source: Front Med (Lausanne). 2022 May 9;9:893273. doi: 10.3389/fmed.2022.893273 (PMC9124890; doi:10.3389/fmed.2022.893273)
Supplement: Supplementary file 1 [file Data_Sheet_1.PDF]

Supplementary Material

Supplementary Table 1

(a)

		**ADAMTS13 activity**	
		<10%	>10%
**PLASMIC score**	6–7	9 patients	1 patient
	0–5	0 patient	14 patients

(b)

**Sensitivity**	100.00%	95% CI, 66.37% to 100.00%
**Specificity**	93.33%	95% CI, 68.05% to 99.83%

(c)

**Positive predictive value**	90.00%	95% CI, 57.53% to 98.35%
**Negative predictive value**	100.00%	

ADAMTS13, a disintegrin and metalloprotease with thrombospondin type 1 repeats, member 13; CI, confidence interval

Supplementary Table 2

(a)

		**ADAMTS13 activity**	
		<10%	>10%
**French score**	2–3	9 patients	5 patients
	0–1	0 patient	10 patients

(b)

**Sensitivity**	100.00%	95% CI, 66.37% to 100.00%
**Specificity**	66.67%	95% CI, 38.38% to 88.18%

(c)

**Positive predictive value**	64.29%	95% CI, 46.81% to 78.64%
**Negative predictive value**	100.00%	

ADAMTS13, a disintegrin and metalloprotease with thrombospondin type 1 repeats, member 13; CI, confidence interval
